# Global mapping of epidemic risk assessment toolkits: A scoping review for COVID-19 and future epidemics preparedness implications

**DOI:** 10.1371/journal.pone.0272037

**Published:** 2022-09-23

**Authors:** Bach Xuan Tran, Long Hoang Nguyen, Linh Phuong Doan, Tham Thi Nguyen, Giang Thu Vu, Hoa Thi Do, Huong Thi Le, Carl A. Latkin, Cyrus S. H. Ho, Roger C. M. Ho

**Affiliations:** 1 Institute for Preventive Medicine and Public Health, Hanoi Medical University, Hanoi, Vietnam; 2 Bloomberg School of Public Health, Johns Hopkins University, Baltimore, MD, United States of America; 3 Karolinska Institutet, Stockholm, Sweden; 4 National Centre For Youth Substance Use Research, University of Queensland, Brisbane, Australia; 5 Institute of Health Economics and Technology, Hanoi, Vietnam; 6 Department of Psychological Medicine, National University Hospital, Singapore, Singapore; 7 Department of Psychological Medicine, Yong Loo Lin School of Medicine, National University of Singapore, Singapore, Singapore; 8 Institute for Health Innovation and Technology (iHealthtech), National University of Singapore, Singapore, Singapore; Neijiang Normal University, CHINA

## Abstract

Preparedness and responses to infectious disease epidemics and pandemics require the understanding of communities’ and multisectoral systems’ characteristics with regards to diseases transmission and population’s vulnerabilities. This study aimed to summarize measurement profiles of existing risk assessment toolkits to inform COVID-19 control at global and national levels. An online search in different databases and online sources was performed to identify all epidemic risk and vulnerability assessment instruments. Medline/PubMed, Web of Science databases, and websites of public health organizations were used for the searching process. Of 14 toolkits, levels of setting were mostly at the global or nation level. Components such as Governance and Legislation, Financing, Health Service Provision, and Human Resources are key domains in almost all toolkits. Some important issues for disease detection and surveillance, such as laboratory or capacity of the community for disease control, were not adequately addressed in several toolkits. Limited studies were found that validated the toolkits. Only five toolkits were used in COVID-19 studies. This study provides a summary of risk assessment toolkits to inform epidemic responses. We call for global and national efforts in developing more contextualized and responsive epidemic risk assessment scales incorporating specific-disease and -country factors to inform operational decisions making and strengthen countries’ capacities in epidemic responses.

## Introduction

Novel coronavirus disease 2019 (COVID-19) has been officially recognized as a global pandemic by the World Health Organization, with more than 523 million positive cases and 6.27 million deaths reported in approximately 220 countries and territories until 19 May 2022 [1]. This widespread transmission is understandable since only half of the states had sufficient preparations for this health emergency, according to a global analysis in 2020 [2]. From 2020 to 2021, the number of cases and deaths still remarkably increase although many efforts have been performed to prevent and control the pandemic in all nations, from accelerating the vaccination coverage, strict quarantine, and preventive measures such as social distancing, contact tracing, face masks mandatory [1]. The presence of the Delta variant and other potential variants have made the governments of countries realize that the "Zero Covid" strategy is completely impossible (except for China) and need to develop strategies for living with COVID-19 in the future [3].

There is no doubt that residents in all countries, regardless of economic conditions, are currently more vulnerable to infectious disease outbreaks [4]. The number of infectious disease epidemics over the last 20 years, at both local and global levels, is far beyond the number of epidemics in the whole last century [5], resulting in remarkable health and economic losses in affected nations [4]. This explosion is attributable to substantial increases in international connectivity, population density, and human-wild animal interactions, as well as amplified by alterations of ecological factors such as climate change or rapid urbanization [6–8]. Notably, in addition to existing periodical infectious diseases (for example, dengue, malaria, or influenza), a diversity of novel disease epidemics with high morbidity and mortality rates have been recorded such as severe acute respiratory syndrome–SARS, H1N1, Zika, Middle East Respiratory Syndrome—MERS or, most recently, COVID-19 [9, 10]. The emergence of all pathogens is unpredictable, but they began with some local cases then spread out to become international crises due to global travel and trade [11]. Therefore, albeit the unpredictability of novel epidemic agents, preparedness in each country is critically important to respond to localized outbreaks, prevent the spread, and mitigate the epidemic’s burden [12].

Preparedness and responses to infectious disease epidemics and pandemics require the understanding of communities’ and systems’ characteristics with regards to diseases transmission and population’s vulnerabilities [4, 12]. Since the International Health Regulations (IHR) was issued (2005) and came into force (2007) [13], there have been substantial efforts to quantify the total risks and assess vulnerabilities of different populations at national, regional, and global levels. For instance, in 2005, Joint External Evaluation Tool (JEE) was developed that adopted the IHR regulations to externally assess the country’s capacity to detect and respond to the public health risks [14]. In 2007, the State Parties Self-Assessment Annual Reporting Tool (SPAR) was also constructed to measure the progress in acquiring IHR targets [15]. The most recent initiatives for risk and vulnerability assessment were the Epidemic Preparedness Index [12], the Health Vulnerability Index for Disaster Risk Reduction [16], the Global Health Security Index (GHSI) [17], and the EpiRisk Tool [18], which use open-source data for national level-gap analysis.

Examples of the use of these assessment toolkits for epidemic preparation have been described in the literature. Espinal et al. analyzed IHR’s core capacities to inform the gaps of Latin America and the Caribbean countries before Ebola outbreaks and suggested that countries should strengthen their capacities and monitoring approaches [19]. Glynn et al. assessed the preparedness of Ireland against Ebola and Zika epidemics, which had occurred in West Africa in 2007–2008, and found that the country had a good preparation before these epidemics [20]. Some instruments have been applied to evaluate national preparedness for COVID-19 outbreaks. For example, Craig et al. analyzed GHSI data of 112 countries and showed that 54/112 countries had scored lower than the global average [21]. The authors also indicated that all Pacific Island countries and territories belonged to the lowest preparedness group for COVID-19 [21]. Another study using SPAR by Kandel et al. revealed that among 182 countries, 24% (44 countries) did not have any effective mechanisms to enable responses to COVID-19 [2]. Wong et al. showed that a higher IHR score was negatively associated with the number of new COVID-19-related cases and deaths [22].

To date, limited studies attempted to have the consensus in accordance with the necessary elements that should be included in the epidemic risk assessment. This study summarized the developmental history, profiles, and applications of existing toolkits for evaluating global risks and vulnerabilities of infectious diseases. Second, it analyzed gaps in evidence and implications of these existing measurements for COVID-19 preparedness and responses at national and global levels.

## Methods

### Search strategy

In this study, we employed two searching strategies for identifying the epidemic risk assessment toolkits: 1) Online search in electronic databases to identify scientific peer-reviewed articles; and 2) Online search in public health organization websites to identify grey literature. First, we conducted the online search in Medline/PubMed and Web of Science databases to identify the peer-reviewed articles published from January 1^st,^ 2000 to June 30^th,^ 2021. The searches strings and results are presented in Table 1. We combined all searches strings by using the Boolean operator “AND”. Second, we searched grey literature on websites of the following organizations: World Health Organization (WHO), the United States Center for Disease Control and Prevention (CDC), the United States Agency for International Development (USAID), European Commission, and the European CDC. We also sought in the references of these selected documents additional eligible publications. The searching process was performed in April 2020 and updated in July 2021.

**Table 1:** Searches strings.

	PubMed/Medline (n = 2575)
**1**	assessment [Title/Abstract] OR measurement [Title/Abstract] OR tool [Title/Abstract] OR toolkit [Title/Abstract] OR checklist[MeSH Terms] OR checklist[Title/Abstract] OR index[Title/Abstract] OR scale[Title/Abstract] OR “risk analysis” [Title/Abstract]
**2**	global [Title/Abstract] OR national [Title/Abstract] OR subnational[Title/Abstract]
**3**	emergencies[MeSH Terms] OR emergencies[Title/Abstract] OR emergency[Title/Abstract] OR disasters[MeSH Terms] OR disasters[Title/Abstract] OR disaster[Title/Abstract] OR pandemics[MeSH Terms] OR pandemics[Title/Abstract] OR pandemic[Title/Abstract] OR “infectious disease” [Title/Abstract] OR “communicable disease” [Title/Abstract] OR infection [Title/Abstract]
**4**	planning[Title/Abstract] OR preparedness[Title/Abstract] OR response[Title/Abstract]
**5**	Human[MeSH Terms]
**6**	(“2000/01/01” [PDAT]:“2021/06/30” [PDAT])
	Web of Science (n = 1139)
**1**	AB = (assessment OR measurement OR tool OR toolkit OR checklist OR index OR scale OR “risk analysis”)
**2**	AB = (global OR national OR subnational)
**3**	AB = (emergencies OR emergency OR disasters OR disaster OR pandemics OR pandemic OR “infectious disease” OR “communicable disease” OR infection)
**4**	AB = (planning OR preparedness OR response)
**5**	TS = (Human)
**6**	Publication date: 2000/01/01–2021/06/30

### Selection criteria

All articles or documents related to the development and use of epidemic risk and vulnerability assessment instruments were included. Other eligibility criteria included: 1) Being published in English; 2) Published from January 1^st^ 2000 to June 30^th^ 2021; 3) Covering international, national or subnational assessment; 4) Presence of checklists, indicators or scales for assessing national epidemic risk and vulnerability. For those published from 2020 to 2021, we also sought publications that used these tools in COVID-19 topics to examine the application of these tools. We excluded papers covering instruments that only focused on specific diseases, hazards which were not infectious diseases or natural reasons (for instance, bioterrorism-related epidemic) such as the World Health Organization Measles Programmatic Risk Assessment Tool, the WHO human health risk assessment toolkit for chemical hazards, or Risk assessment guidelines for diseases transmitted on aircraft, or Joint European Pandemic Preparedness Self Assessment Indicators (focusing on influenza), which might not be widely applied for other conditions. Civil emergency assessments were excluded if they did not cover epidemic risk assessment or preparedness or response. We also excluded papers that were 1) narrative review, systematic review or meta-analysis; 2) abstract, study protocol, conference paper, conference proceedings, news, letters and others that were not scientific articles or organization’s reports or guidelines (if they mentioned epidemic risk assessment scales).

### Data extraction and synthesis

Extracted information included the name of instruments, year of the first publication, origin, sources of data for assessment, number of domains, name of domains, number of items, and score range. We identified whether an instrument was correlated with each other (concurrent validity) by extracting the data of correlation measures (for example, Pearson or Spearman correlation coefficient, regression coefficient, odds ratio, relative risk, or hazard ratio). Information about the application of these tools in the COVID-19 pandemic was extracted in publications reporting these topics of interest. Two independent researchers performed the data extraction. Disagreements were solved by the third senior researcher. Table 2 described the evaluation framework used to compare different assessment tools, which were adapted from previous reviews [23–25].

**Table 2:** Evaluation framework.

Criteria	Description
Source of origin	Organizations or individuals who developed the tool
Objective and topics	The purposes of the tool, including assessment of risk and preparedness for epidemic and/or natural disasters
Source of data/utility	Extend to which the tool was measured and reported
Completeness	Number and name of dimensions/criteria and whether these tools were periodically assessed or not.
Clarity of measurement	Extend to which the manners to measure the items/indicators/parameters were described in the tool.
Validity of measurement	Extend to which the tool’s score was correlated with other tools (criterion validity), correlated with other epidemic outcomes and responses (construct validity), and correlated with COVID-19 pandemic outcomes (predictive validity)
Scope	Extend to which the data was applied for, for example, international, national, or community level.
Feasibility	The number of items/indicators, score range and interpretability. User-friendly was also evaluated.
Specification of an accountable entity	Extend to which the tool determined which organizations or individuals were responsible for reporting and completing the tool.
Strengths and limitations	Characteristics of the tools that were described in the tool and in the literature which can be useful or barriers for their applications in decision making.

## Results

**Fig 1:**
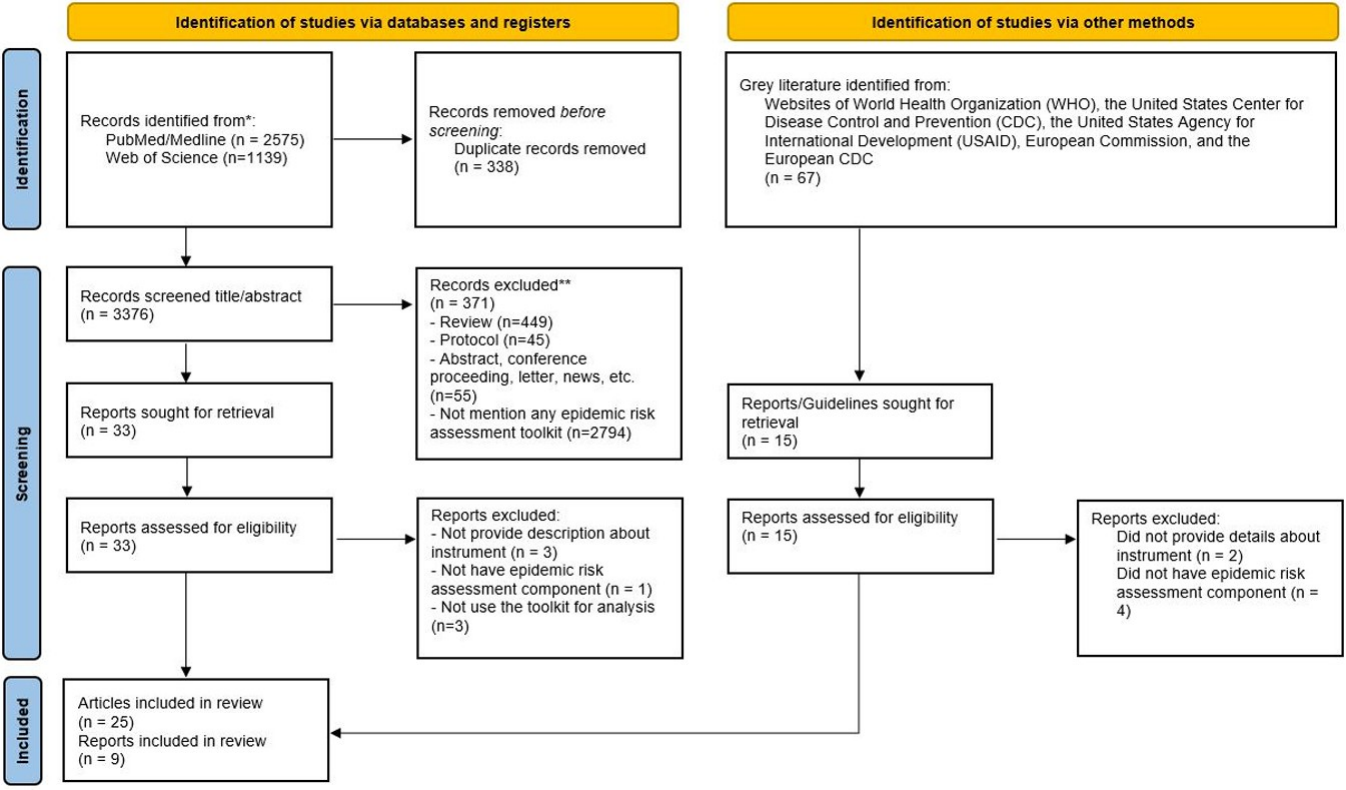
Results of the searching process.

Overall, 3714 articles and 67 grey literatures were identified through the searching process. After the screening stage, a total of 34 documents (25 articles and 9 reports) that used 14 toolkits were found. **Fig 1.** illustrated the searching process.

**Table 3** provides a list of the features of each toolkit. The Joint External Evaluations (JEE) instrument was the first toolkit developed after the issue of the IHR framework in 2005 [14], following by the Self-Assessment Annual Reporting (SPAR) in 2007. These two toolkits adapted the IHR conceptual frameworks in measuring the risk and vulnerability of each country to epidemic [15]. The most recent instrument was the EpiRisk Tool, which was developed to evaluate the potential severity of an infectious epidemic in a country [18]. Most of the toolkits were developed by the United States organizations (5/14) [12, 26–30], following by European institutions (for example England or European Centre for Disease Prevention and Control) [31–34] and international organizations (e.g. World Health Organization) [14, 15]. The number of criteria/dimensions in each instrument ranged from 2 to 14 with the number of indicators/items ranging from 7 (for Threat and Hazardous Incident Risk Assessment (THIRA) [27] to 140 (for Global health security index) [29]. Levels of setting were usually at a global or national level, while only several scales have been developed specifically to serve the community levels (such as THIRA, US CDC Public Health Preparedness Capabilities and the CDC’s Social Vulnerability Index). Notably, only 5/14 instruments had periodical assessments; for example, JEE, SPAR, US CDC Public Health Preparedness Capabilities, and Cambridge Global Risk Index had annual reports while the CDC’s Social Vulnerability Index was biannually assessed.

**Table 3:** General profiles of risk assessment instruments.

No	Instrument	Year of publication	Country/ Organization of Origin	Sources of data	No. of criteria/ dimensions	Name of criteria/dimensions	No. of items/ indicators	Score range (and cutoffs)	Periodical assessment
1	Joint External Evaluations [14]	2005	World Health Organization	Self-reported data from countries’ survey	19	1) National legislation, policy and financing	47	From 1 to 5:	Annually
						2) IHR coordination, communication and advocacy		1 = No capacity; 2 = Limited capacity; 3 = Developed capacity; 4 = Demonstrated capacity; 5 = Sustainable capacity	
						3) Antimicrobial resistance			
						4) Zoonotic disease			
						5) Food safety			
						6) Biosafety and biosecurity			
						7) Immunization			
						8) National laboratory system			
						9) Surveillance			
						10) Reporting			
						11) Human resources (animal and human health sectors)			
						12) Emergency preparedness			
						13) Emergency response operations			
						14) Linking public health and security authorities			
						15) Medical countermeasures and personnel deployment			
						16) Risk communication			
						17) Points of entry			
						18) Chemical events			
						19) Radiation emergencies			
2	Self-Assessment Annual Reporting (SPAR) [15]	2007	World Health Organization	Online annual self-reporting	13	1) Legislation and financing	24	From 1 to 5	Annually
						2) IHR coordination and National Focal Point functions		1 = Policies/strategies are not available	
						3) Zoonotic events and the human-animal interface		2 = Policies/strategies are available in national level	
						4) Food safety		3 = Policies/strategies are available in all relevant sectors	
						5) Laboratory			
						6) Surveillance			
						7) Human resources		4 = Policies/strategies are available in national, intermediate and local levels by all relevant sectors	
						8) National health emergency framework		5 = Policies/strategies are updated frequently	
						9) Health service provision			
						10) Risk communication			
						11) Points of entry (PoEs)			
						12) Chemical events			
						13) Radiation emergencies			
3	CDC’ Social Vulnerability Index [26]	2011	United States Center for Disease Control and Prevention	American Community Survey	4	1) Socio-economic status;	15	From 0 “lowest level of social vulnerability” to 1 “highest level of social vulnerability”	Biannual
						2) Household Composition & Disability;			
						3) Minority Status & Language;			
						4) Housing Type & Transportation			
4	US CDC Public Health Preparedness Capabilities [30]	2011	United States Center for Disease Control and Prevention	Self-reporting	6	1) Community Resilience	15	None of scoring system	Annually
						2) Incident management			
						3) Information management			
						4) Countermeasures and Mitigation			
						5) Surge Management			
						6) Biosurveillance			
5	Generic preparedness planning for public health emergencies [34]	2011	European Commission	Self-reporting	7	1) Information management	47	None of scoring system	N/A
						2) Communication			
						3) Scientific/Evidence-based advice			
						4) Health crisis management structures			
						5) Health sector preparedness			
						6) Intersectoral collaboration			
						7) Management of plans			
6	Threat and Hazardous Incident Risk Assessment (THIRA) [27]	2012	United States Department of Homeland Security	Online and others national and sub-national reports	5	1) Prevention	7	None of scoring system	N/A
						2) Protection			
						3) Mitigation			
						4) Response			
						5) Recovery			
7	Infectious Disease Vulnerability Index [28]	2015	Research and Development (RAND) Corporation, United States	Secondary data from WHO, World Bank, publications	7	1) Demographics	48	From 0 “highest vulnerability” to 1 “lowest vulnerability”	N/A
						2) Health care			
						3) Public health			
						4) Disease dynamics			
						5) Political domestics			
						6) Political-international			
						7) Economics			
8	INFORM Epidemic Risk Index [31]	2018	European Union	Self-reporting	3	1) People at risk	89	From 0 “lowest risk” to 10 “highest risk”	N/A
						2) Vulnerability			
						3) Lack of coping capacity			
9	INFORM Epidemic Global Risk Index [32]	2018	European Union	Self-reporting	3	1) Hazards & exposure	100	From 0 “lowest risk” to 10 “highest risk”	N/Á
						2) Vulnerability			
						3) Lack of coping capacity			
10	Cambridge Global Risk Index [33]	2018	Cambridge Centre for Risk Studies, England	Secondary data from multiple sources	5	1) Natural Catastrophe and Climate	21	• Expected loss: total gross domestic production (GDP) loss and percentage GDP loss	Annually
						2) Finance, Economics and Trade		• Threat analysis: from 1“small threat” to 3 “large threat”	
						3) Geopolitics and Security		• City recoverability: from 1 “very strong” to 5 “very weak”	
						4) Technology and Space			
						5) Health and Humanity			
11	Health Vulnerability Index for Disaster Risk Reduction [16]	2019	China	Secondary data from WHO, World Bank, publications	3	1) Population status	9	From 1 “lowest vulnerability” to 5 “highest vulnerability”	N/A
						2) Disease prevention			
						3) Coping capacity			
12	Epidemic Preparedness Index [12]	2019	United States	Secondary data from multiple sources	5	1) Public Health Infrastructure	23	From 0 “Least preparedness” to 100 “Most preparedness”	N/A
						2) Physical Infrastructure			
						3) Institutional Capacity			
						4) Economics Resources			
						5) Public health communication			
13	Global health security index [17]	2019	Nuclear Threat Initiative, Johns Hopkins Bloomberg School of Public Health, United States	Public data sources from individual countries and international organizations	6	1) Prevention	140	From 0 “Least preparedness” to 100 “Most preparedness”	N/A
						2) Detection and reporting			
						3) Rapid response			
						4) Health system			
						5) Compliance with international norms			
						6) Risk environment			
14	EpiRisk [18]	2020	Indonesia	Secondary data from WHO, CDC, World Bank, Peace Institute, grey literature, publications	2	1) Disease-related parameters: Disease pathogen, basic reproductive number, mode of transmission, asymptomatic transmission, case fatality rate, therapy/drug availability, vaccine availability	14	From 1 to 42, with low risk (<21), moderate risk (21–29), high risk (30–37) and extreme risk (38–42)	N/A
						2) Country-related parameters: Income, total health expenditure (% gross domestic product), peace index, land border, population density, physician density, hospital beds			

Note: N/A: not available; WHO = World Health Organization; CDC = Center for Disease Control and Prevention.

**Table 4** indicates the breaths of 14 selected measures. In this study, we classified the contents or indicators of selected instruments into three major groups: system components, demographic and community components, and other specific components. Overall, none of the toolkits fully covered all components. The CDC’s Social Vulnerability Index [26] covered the least components given that this tool only measured some indicators such as poverty, unemployed, income, education, age, housing or transportation. Meanwhile, the global health security index [17] covered the highest number of components because 140 indicators were used to estimate this index. As for the breadth of measurement, components such as Governance and Legislation, Financing, Health Service Provision, and Human Resources are key domains in almost all toolkits. Meanwhile, we found that some important issues for disease detection and surveillance, such as laboratory or capacity of the community for disease control, were not adequately addressed in several toolkits.

**Table 4:** Coverage of selected instruments.

Instrument	System components														Demographic and community components						Specific components							
	International Health Regulations principles	Governance and Legislation	Stalkholder coordination	International Collaboration	Plans/strategies for preparedness and response	Financing	Human resources for health	Other resources	Healthcare infrastructure	Laboratory	Surveillance	Health service provision	Reporting system and network	Risk communication and management	Demographic characteristics	Household characteristics	Community characteristics	Transportation and mobility	Literacy/KAP	Resilience and recovery	Antimicrobial resistance	Diseases transmission	Food security and safety	Chemical and radiation	Biosafety and biosecurity	Climate & Natural disasters	Points of entry	Others
1. Joint External Evaluations [14]	x	x	x		x	x	x	x	x	x	x	x	x	x							x	x	x	x	x		x	
2. Self-Assessment Annual Reporting (SPAR) [15]	x	x	x		x	x	x	x	x	x	x	x		x								x	x	x			x	
3. CDC’ Social Vulnerability Index [26]															x	x		x										
4. US CDC Public Health Pre4. paredness Capabilities [30]			x		x		x	x	x	x	x	x	x	x	x	x	x	x	x	x	x	x	x	x	x	x	x	x
5. Generic preparedness planning for public health emergencies [34]	x	x	x	x	x		x	x	x	x	x	x	x	x								x	x	x		x	x	x
6. hreat and Hazardous Incident Risk Assessment (THIRA) [27]			x		x	x	x	x	x	x	x	x		x		x		x		x								
7. Infectious Disease Vulnerability Index [28]	x	x	x	x	x	x	x	x	x			x	x	x	x		x	x	x	x		x				x		x
8. INFORM Epidemic Risk Index [31]	x	x			x	x	x	x	x			x			x	x	x		x			x	x			x	x	
9. INFORM Epidemic Global Risk Index [32]	x	x			x	x	x	x	x			x			x	x	x	x	x			x	x			x	x	x
10. Cambridge Global Risk Index [33]		x				x						x		x						x		x	x	x	x	x		x
12. Health Vulnerability Index for Disaster Risk Reduction [16]							x		x			x			x		x					x						
13. Epidemic Preparedness Index [12]		x	x	x		x	x	x	x		x	x	x	x			x	x	x									
13. Global health security index [17]	x	x	x	x	x	x	x	x	x	x	x	x	x	x		x	x	x	x	x	x	x	x	x	x	x	x	x
14 EpiRisk [18]		x				x	x		x			x			x		x					x						

* KAP = Knowledge-attitude-practice.

**Table 5** depicts the usages, properties, strengths and limitations of the selected toolkits. All toolkits determined that they covered the national level except CDC’s Social Vulnerability Index. Six of the instruments measured preparedness in epidemic and/or natural disasters (in which epidemic risk assessment was one of the components), and others measured the risk of epidemic. Only the validity of 7/14 instruments was evaluated. Epidemic Preparedness Index toolkit was found to have the strongest correlations with JEE (r = 0.85–0.86 [12, 35]) and SPAR (r = 0.62 [12]), following by Global health security index (JEE: r = 0.82 [36]), and INFORM Epidemic Risk Index toolkit (SPAR: r = 0.47 [37] and JEE: r = 0.6 [37]).

**Table 5:** Usage, properties, strengths and limitations of risk assessment tools.

Instrument	Objectives	Topics of usage	Scope	Strengths	Limitations	Validity	
						Construct validity	Criterion validity
1. Joint External Evaluations [14]	Assess the capacity of a country in preventing, detecting and responding an acute/emerging public health events according to the International Health Regulations (IHR)	Preparedness in epidemic and natural disasters	National	1) IHR-based assessment	Only data of more than 90 countries were available	SPAR: r = 0.57 [38]	Correlated with quality of outbreak responses in Ethiopia, Nigeria and Madagascar [39]
				2) Refined criteria		EPI: r = 0.85–0.86 [12, 35]	
				3) External validation and objective measurement		IDVI: r = 0.64 [37]	
						IERI: r = 0.6 [37]	
				4) Clear and simple		GHSI: r = 0.82[36]	
2. Self-Assessment Annual Reporting (SPAR) [15]	Assess the IHR capacities needed in detecting, evaluating, notifying, reporting and responding to public health risk and acute events	Preparedness in epidemic and natural disasters	National	1) IHR-based assessment	Self-report data	JEE: r = 0.57 [38]	N/A
				2) Refined criteria		EPI: r = 0.62 [12]	
				3) Clear and simple		IDVI: r = 0.46 [37]	
						IERI: r = 0.47 [37]	
3. CDC’ Social Vulnerability Index [26]	Evaluate the relative vulnerability of communities to detect places that need support to respond acute events such as disasters and disease outbreaks	Risk of epidemic and natural disasters	Community	1) Annual updated	1) Weak correlation with health outcomes	N/A	N/A
				2) Combine geospatial information for measurement	2) Measures such as minority status might be skewed.		
				3) Refined criteria	3) Results of some sub-population groups should be carefully interpreted.		
					4) Use census data which might lead to potential bias		
4. US CDC Public Health Preparedness Capabilities [30]	Checklist to measure the preparedness off national and local public health systems for emergency including epidemics	Preparedness in epidemic and other hazards	National, community	1) Annual updated	1) Subjective evaluation	N/A	N/A
				2) Comprise community preparedness	2) None of the scoring system for comparison/quantitative measurements		
				3) Very details in each task	3) Lack of qualitative evaluation		
				4) Clear and simple	4) No validation		
5. Generic preparedness planning for public health emergencies [34]	Checklists to evaluate the necessary requirements for minimum public health preparedness in emergency conditions	Preparedness in epidemic and other hazards	International, national	1) Cover many aspects in preparedness	1) Subjective evaluation	N/A	N/A
				2) Helpful task list	2) None of the scoring system for comparison/quantitative measurements		
				3) Clear description in each task	3) Lack of qualitative evaluation		
					4) No validation		
					5) Plain text		
6. Threat and Hazardous Incident Risk Assessment (THIRA) [27]	Evaluate risks and associated impacts	Risk of natural and unnatural hazards and disasters	National, community	Refined criteria	1) Subjective evaluation	N/A	N/A
					2) None of scoring system for comparison/quantitative measurements		
7. Infectious Disease Vulnerability Index [28]	Detect the most vulnerable countries to infectious disease epidemics	Risk of epidemic	National	1) Objective data from different sources	1) Depend on availability and accuracy of data	IERI: r = 0.86 [37]	
				2) High availability of indicators that improve the comparability across nations	2) Unable to predict the occurrence of outbreaks	SPAR: r = 0.46 [37]	
						JEE: r = 0.64 [37]	
				3) Refined criteria			
8. INFORM Epidemic Risk Index [31]	Assess national and community’s vulnerabilities and risks to epidemic	Risk of epidemic	National, community	1) Objective data from different sources	1) Not cover the indicators of IHR and JEE	IDVI: r = 0.86 [37]	
						SPAR: r = 0.47 [37]	
				2) High availability of indicators that improve the comparability across nations	2) Not include immunization rate and diagnosis capacity	JEE: r = 0.6 [37]	
					3) Depend on availability and accuracy of data		
9. INFORM Epidemic Global Risk Index [32]	Incorporate epidemic assessment into INFORM global risk index model to measure the risk of different crises	Risk of epidemic, hazards and disasters	National	1) Objective data from different sources	1) Not cover the indicators of IHR and JEE	N/A	N/A
				2) High availability of indicators that improve the comparability across nations	2) Not include immunization rate and diagnosis capacity		
				3) Refined criteria	3) Depend on availability and accuracy of data		
10. Cambridge Global Risk Index [33]	Estimate risk and consequences of different threats on worlds’ economy	Risk of epidemic, hazards and disasters	National	1) Objective data from different sources	1) Depend on availability and accuracy of data	N/A	N/A
				2) High availability of indicators that improve the comparability across nations	2) Limits in covered outbreaks		
11. Health Vulnerability Index for Disaster Risk Reduction [16]	Evaluate the health risk of hazards and disasters at the national level	Risk of epidemic, hazards and disasters	National	1) Objective data from different sources	1) Not include sociodemographic and political vulnerability aspects	N/A	N/A
				2) High availability of indicators that improve the comparability across nations	2) Depend on availability and accuracy of data		
					3) Limits in covered disasters		
12. Epidemic Preparedness Index [12]	Measure national preparedness (detect and respond) to future disease outbreaks	Preparedness to respond to epidemics	National	Able to update quickly the change of countries	1) Data about availability of response plans and public trust in government could not capture	JEE: r = 0.85–0.86 [12, 35]	Positive associations with the timeliness of epidemic detection and reporting and vaccination rates across countries
						SPAR: r = 0.62 [12]	
					2) Not cover disease-specific elements		
					3) Depend on availability and accuracy of data		
13. Global health security index [17]	Evaluate health security and capacities to respond to infectious disease epidemics	Risk of epidemics, preparedness to respond	National	1) Large number of indicators	1) Results were skewed to high-income countries [40]	JEE: r = 0.82 [36]; rh0 = 0.82 [41]	Deaths from communicable disease (rh0 = -0.56) [41]
				2) Objective data from different sources	2) Some countries may be underestimated due to unavailable data [38]		
				3) High availability of indicators that improve the comparability across nations	3) Inconsistent scoring system		
					4) Questioned validity of some indicators [40]		
14. EpiRisk [18]	Evaluate potential severity of disease outbreaks	Risk of epidemics	National	1) Simple and rapid assessment tool	1) Depend on availability and accuracy of data	N/A	Positive association with severity of outbreaks across countries [18] (through death cases reported)
				2) Objective data from different sources	2) Limits in covered diseases		
				3) High availability of indicators that improve the comparability across nations	3) Data were collected from various sources with different time points, leading to potential biases		
				4) Combine both disease and country parameters			

Note: N/A: not available; r: correlation coefficient; IHR = International Health Regulations; JEE = Joint External Evaluations; SPAR = Self-Assessment Annual Reporting; IDVI = Infectious Disease Vulnerability Index; IERI = INFORM Epidemic Risk Index; GHSI = Global health security index.

Only 5/14 toolkits (JEE, SPAR, SVI, IDVI, and GHSI) were found to be used in COVID-19 related studies in the searching period. The main findings of these studies are summarized in **Table 6**. Only one study showed that JEE score was poorly related to the COVID-19 mortality rates [36]. Several studies on IDVI and GHSI had similar findings when showing that scores of these tools had low or no correlation with COVID-19 outcomes [36, 42–44]. GHSI was argued to be potential in projecting subnational responses to COVID-19 [45]. Other toolkits such as SPAR, SVI showed negative associations with COVID-19 incidence and mortality rates [17, 22, 46–50].

**Table 6:** Application of selected instruments in COVID-19 risk assessment.

Instrument	Correlations			Other findings
	Risk of COVID-19	Preparedness and response	COVID-19 clinical outcomes	
1. Joint External Evaluations (JEE)	N/A	N/A	• JEE had weak correlations with COVID-19 mortality rates [36]	N/A
2. Self-Assessment Annual Reporting (SPAR)	• Countries with the highest level of importation risk had moderate-to-high SPAR scores, while countries with moderate risk had variable SPAR scores [37].	N/A	• Negatively associated with the number of new COVID-19-related cases and deaths per 100,000 population within 30 days [22]	• SPAR scores of Japan, Iran, South Korea, the United Kingdom and the United States were above global and regional averages [51]
				• 56% of countries readied in having effective responses to public health crises, 57% countries readied in preventing, detecting and controlling the outbreaks [2].
3. CDC’ Social Vulnerability Index (SVI)	N/A	• Higher SVI was positively associated with increased COVID-19 testing [49]	• Higher SVI was positively associated with increased COVID-19 cases and deaths, particularly ethnic minorities and disadvantaged household conditions [17, 46–50]	N/A
4. US CDC Public Health Preparedness Capabilities	N/A	N/A	N/A	N/A
5. Generic preparedness planning for public health emergencies	N/A	N/A	N/A	N/A
6 Threat and Hazardous Incident Risk Assessment (THIRA)	N/A	N/A	N/A	N/A
7 Infectious Disease Vulnerability Index (IDVI)	• Countries with the highest level of importation risk had moderate-to-high IDVI scores. Countries with moderate risk had low IDVI scores [37].	N/A	• IDVI had low power in predicting COVID-19 cases and deaths [42]	N/A
8. INFORM Epidemic Risk Index	N/A	N/A	N/A	N/A
9. INFORM Epidemic Global Risk Index	N/A	N/A	N/A	N/A
10. Cambridge Global Risk Index	N/A	N/A	N/A	N/A
11. Health Vulnerability Index for Disaster Risk Reduction	N/A	N/A	N/A	N/A
12. Epidemic Preparedness Index (EPI)	N/A	N/A	N/A	N/A
13. Global health security index (GHSI)	N/A	N/A	• GHSI had weak correlations [36], or no correlation with COVID-19 mortality rates [43, 44]	• 54/112 countries had scored lower than the global average [21]
			• GHSI was positively related to total cases and deaths per million [45]. GHSI had the potential in predicting outcomes of local response.	• GHSI scores of Japan, South Korea, the United Kingdom and the United States were above global and regional averages [51]. GHSI score of Iran was below the global average [51].
			• GHSI overestimated the preparedness of OECD countries with high GHSI scores; and underestimated the preparedness of OECD countries with low GHSI score [52]	• All Pacific islands countries where data were available belonged to the “least preparedness” group [21].
14. EpiRisk	N/A	N/A	N/A	N/A

Abbreviation: IHR = International Health Regulations; JEE = Joint External Evaluations; SPAR = Self-Assessment Annual Reporting; IDVI = Infectious Disease Vulnerability Index; GHSI = Global health security index; SVI = Social Vulnerability Index; OECD = Organization for Economic Cooperation and Development.

## Discussion

Monitoring the risk and vulnerability of different communities and countries is critically important for national preparedness and responses to the epidemic. This study summarized the profile and developmental history of toolkits, as well as reveals diverse breadths of measurements and their applications in various levels of administration.

The current paper revealed that fourteen toolkits have been developed to assess the risk of infectious disease epidemics, but most of them lacked validation and were not widely applied. Indeed, before 2020, only seven toolkits were assessed their measurement properties including JEE, SPAR, IDVI, INFORM Epidemic Risk Index, Epidemic Preparedness Index, Global health security index and EpiRisk, raising questions about the applicability of these toolkits in planning response strategies. Prior literature emphasized that although the GHSI was considered a comprehensive evaluation with 140 items, its validity and reliability are questionable, which could lead to miscalculations and misinterpretations [40].

In terms of content, the scales cover quite adequately the aspects related to susceptibility to the disease, but none of the scales can fully cover the whole range of key factors. Most of the instruments use data from a national report or global databases to mirror the policymaking and health care performance. However, contextualized or specific-country factors, which play a critical role in predicting the risk of and response to infectious diseases epidemic [2], are lacking. Lessons learned from the COVID-19 epidemic and other previous outbreaks suggested that the IHR framework has weaknesses in its ability to guide countries to respond to the pandemic [13]. For instance, Kaiser et al. indicated that GHSI had poor predictability and did not meet the need of policymakers in the community [44]. Similarly, Ye Ji et al. found that GHSI was not helpful in assessing the preparedness and response of a country against global pandemic [45]. Although fundamental for developing epidemic risk assessment toolkits, some critical materials are not completely complied by all country members of the World Health Organization for several reasons such as insufficient resources for following recommendations, or unwillingness to change systems following the World Health Organization’s notification of outbreaks due to economic losses [13], resulting in the delay in responses to the epidemic. Another example of the weakness of current toolkits is the highest burden of COVID-19 of the United States and European countries, although these nations were among countries with the highest level of health security and epidemic preparedness in all measures [2, 28, 29]. To date, these countries have been devastated by this epidemic, with the highest number of cases and deaths [1].

It is undeniable that substantial progress among countries has been made by identifying and fulfilling the preparedness gaps via risk assessment toolkits (as shown in previous epidemics such as SARS-CoV, H1N1, Ebola, or Zika) [53]. However, to control the new epidemic as COVID-19, it is necessary to take a holistic view of the available data to identify the current risk, as well as collect more contextualized data (such as individual’s knowledge-practice, behaviors, or trust), and proceed to assess risks not only at the national level but also at the community levels [12]. Some efforts have been performed to improve the credibility of the toolkits. For example, Cartaxo et al. integrated indicators of IDVI and contextualized indicators from other databases such as WHO, World Bank and Brazilian Geography and Statistics Institute to enhance the capability in determining the risk of COVID-19 [54]. The authors found a set of 18 indicators with high sensitivity with only 50% of indicators from IDVI [54]. Indeed, the role of core societal behaviors such as networking is critical in disease transmission during the pandemic. Understanding how people perceive the pandemic, how they perform their social behaviors, as well as how socio-environmental factors impact their behaviors are important and should be integrated into the toolkits for effective control [55].

One important observation of pandemic preparedness and control globally was that countries had very different policies and practice. Although the purpose of developing and applying these toolkits was to inform early response at local, national, and global level, the COVID-19 pandemic disproportionately hit every community that suggested the importance of contextual factors to be addressed. In fact, we have found substantial gap in contextual tools and their limited inclusion of contextual factors in the risk assessment tools. Adapting, validating, and routinely improving the validity of these toolkits are important process for diseases control and prevention.

The findings of this study suggested implications for improving the current toolkits as well as proposed new areas for developing new toolkits for COVID-19 control. First, inadequate evidence about the validation of risk assessment toolkits suggests the need for further studies to validate these toolkits to the standard measures such as JEE and SPAR. This information is critically important to ensure that the toolkit can at least reflect the fundamental dimensions of the IHR framework. Second, since limited toolkits have been used for periodical measures, developing mechanisms to facilitate the use of other toolkits in evaluating the change of global, regional, and national preparedness to further epidemics and pandemics, as well as the gaps that should be filled to strengthen the capacity in epidemic responses. Third, further studies are warranted to develop specific domains for each country’s priorities, given that the majority of the current toolkits might be relatively broad and not reflect important factors within each country. For example, some important variables for epidemic preparedness, such as community variables (for example, population density, community interactions, or coverage of different protective measures) and individual factors (for instance, knowledge-attitude-practice, social network, or public trust in the government) should be included and measured.

This study had several limitations. First, the searching procedure involved only accessible databases, including Medline/PubMed and Web of Science, and purposively selected sources, such as websites of international organizations, thus, might not fully cover those materials which were i) not written in English, ii) not published or indexed, and iii) internal or local use. However, Medline/PubMed is currently the most comprehensive database for life science and biomedical fields with more than 33 million articles [56], and the Web of Science database contained top-quality papers in more than 21,000 peer-reviewed journals in different disciplines [57]. We also performed searches on the websites of different prestigious public health organizations to identify the current epidemic risk and vulnerability assessment instruments. We believed that with our searching strategy, the searching results were credible in covering most of the necessary articles. Second, we did not search the documents that were published in preprint databases such as Medrxiv because they were not peer-reviewed and could have major alterations after completing their review process. Nonetheless, these databases should be considered in further studies to identify potential valuable toolkits for epidemic risk assessment.

## Conclusion

The findings of this study indicated the gaps in evidence on the validity and responsiveness of current risk assessment toolkits as well as the inclusion of population and contextual factors in quantifying epidemic risks. We call for global and national efforts in developing more contextualized and responsive epidemic risk assessment scales incorporating specific-disease and -country factors to inform operational decisions making and strengthen countries’ capacities in epidemic responses.

## Supporting information

S1 Checklist Preferred Reporting Items for Systematic reviews and Meta-Analyses extension for Scoping Reviews (PRISMA-ScR) checklist. (DOCX)
